# Genetic Variation for Nitrogen Use Efficiency Traits in Global Diversity Panel and Parents of Mapping Populations in Pearl Millet

**DOI:** 10.3389/fpls.2021.625915

**Published:** 2021-02-04

**Authors:** Vijayalakshmi Pujarula, Madhu Pusuluri, Srikanth Bollam, Roma Rani Das, Rambabu Ratnala, Gopikrishna Adapala, Vishnukiran Thuraga, Abhishek Rathore, Rakesh K. Srivastava, Rajeev Gupta

**Affiliations:** International Crops Research Institute for the Semi-Arid Tropics (ICRISAT), Patancheru, India

**Keywords:** pearl millet, nitrogen use efficiency, genotypic variations, phenotyping, nitrogen-responsive

## Abstract

Nitrogen (N) is one of the primary macronutrients required for crop growth and yield. This nutrient is especially limiting in the dry and low fertility soils where pearl millet [*Pennisetum glaucum* (L.) R. Br] is typically grown. Globally, pearl millet is the sixth most important cereal grown by subsistence farmers in the arid and semi-arid regions of sub-Saharan Africa and the Indian subcontinent. Most of these agro-ecologies have low N in the root zone soil strata. Therefore, there is an immense need to identify lines that use nitrogen efficiently. A set of 380 diverse pearl millet lines consisting of a global diversity panel (345), parents of mapping populations (20), and standard checks (15) were evaluated in an alpha-lattice design with two replications, 25 blocks, a three-row plot for 11 nitrogen use efficiency (NUE) related traits across three growing seasons (Summer 2017, Rainy 2017, and Summer 2018) in an N-depleted precision field under three different N levels (0%-N_0_, 50%-N_50_, 100%-N_100_ of recommended N, i.e., 100 kg ha^–1^). Analysis of variance revealed significant genetic variation for NUE-related traits across treatments and seasons. Nitrogen in limited condition (N_0_) resulted in a 27.6 and 17.6% reduction in grain yield (GY) and dry stover yield (DSY) compared to N_50_. Higher reduction in GY and DSY traits by 24.6 and 23.6% were observed under N_0_ compared to N_100_. Among the assessed traits, GY exhibited significant positive correlations with nitrogen utilization efficiency (NUtE) and nitrogen harvest index (NHI). This indicated the pivotal role of N remobilization to the grain in enhancing yield levels. Top 25 N-insensitive (NIS-top grain yielders) and N-sensitive (NS-poor grain yielders) genotypes were identified under low N conditions. Out of 25 NIS lines, nine genotypes (IP 10820, IP 17720, ICMB 01222-P1, IP 10379, ICMB 89111-P2, IP 8069, ICMB 90111-P2, ICMV IS89305, and ICMV 221) were common with the top 25 lines for N_100_ level showing the genotype plasticity toward varying N levels. Low N tolerant genotypes identified from the current investigation may help in the identification of genomic regions responsible for NUE and its deployment in pearl millet breeding programs through marker-assisted selection (MAS).

## Introduction

Global nitrogen (N) demand, one of the most expensive farm inputs currently stands at about 117 million metric tons with a projected annual increase of ∼1.5% in the future (FAO, 2019). Indian agriculture consumes over 17 million tons of N fertilizer per year. However, plants can utilize only 30–40% of the applied N for food production and the remaining (up to 60%) is lost to the environment by leaching, de-nitrification, and runoff. Surplus nitrogen pollutes freshwater streams and air which is hazardous to the majority of living species (Hickman et al., 2014; Russo et al., 2017).

Besides, excess usage of N fertilizer not only decreases the efficiency of nutrient use but also affects the rate of economic returns per unit of chemical fertilizer applied. The effect of negative environmental and economic impacts could be reduced through better agronomic practices and also by utilizing N efficient lines with improved nitrogen use efficiency (NUE) (Raun et al., 1999). Hence, improving the NUE of crop plants could help in reducing fertilizer input, increased productivity and profitability coupled with a reduced negative impact on the environment.

Globally, pearl millet (*Pennisetum glaucum* (L.) R. Br) widely grown for food and fodder is considered as the sixth most important cereal crop in terms of area. It is one of the oldest cultivated cereal crops, originated in Africa but later spread to many countries (D’Andrea and Casey, 2002; Manning et al., 2011). This millet crop is vital for food and nutritional security for the world’s poorest people living in different agro-ecological zones. Besides, pearl millet has a rich nutritional profile, wide genetic diversity, high photosynthetic rates being a C_4_ and also provides a healthy balanced diet, thus contributes to the economic security of poor farmers (Srivastava et al., 2019). In the Indian agriculture scenario, pearl millet is grown by poor and marginal farmers under low fertile and rainfall dependent areas often facing drought during different stages of crop growth. From the past few years, pearl millet demand has been increased for its nutritional characteristics and its adaptability to a wide range of climatic conditions (Tako et al., 2015). Since then farmers started applying a high dose of N fertilizer for maximizing grain yield (GY), but excessive usage of N fertilizer leads to low NUE of the crop along with various environmental hazards. In some parts of India, poor farmers lack knowledge about the ideal dose of fertilizer to harvest the real yield potential. Hence, yield improvement of pearl millet under low nitrogen input is indeed beneficial for economic and environmentally sustainable cultivation.

Nitrogen use efficiency is a complex trait, which is associated with various morphological, physiological, molecular, and biochemical changes in plants throughout the life cycle. For a clear understanding of this complex nature, studies of various physiological traits and their close correlation with one or more economically important traits like GY is foremost critical. Nevertheless, it will help in selecting low N tolerant/high yielding lines at different N conditions (Monostori et al., 2016). In general, plant function is always associated with chlorophyll content, which directly indicates the N status of the leaf (Yang et al., 2014). Leaf chlorophyll content and photosynthetic capacity are appropriate benchmarks for identifying high NUE (HNUE) genotypes under low N conditions during field trials (Vijayalakshmi et al., 2015; Kiran et al., 2016). Leaf N status was usually measured by using a hand-held optical chlorophyll meter to monitor the leaf nitrogen status and chlorophyll content. Moreover, leaf area (LA) is also one of the important physiological traits, plays a critical role in enhancing plant biomass (Gianquinto et al., 2004; Monostori et al., 2016).

Nitrogen use efficiency is defined as the ability to produce GY per unit N available in the soil. NUE mainly depends on the results of two main processes, such as N uptake efficiency (NupE), and nitrogen utilization efficiency (NutE) (Good et al., 2004; Hakeem et al., 2012; Vijayalakshmi et al., 2013). NupE is the ability of the plant to take up N from the soil and NutE is the ability to use N to produce gain yield (Ladha et al., 1998; Hirel et al., 2007). Studies on genotypic variations under low and recommended N for NUE traits at both seedling and maturity stage of plants under controlled and field conditions in various crops resulted in the identification of high NUE genotypes possessing high yield sustainability under low N condition (Muchow, 1998; Inthapanya et al., 2000; Le Gouis et al., 2000; Presterl et al., 2003; Anbessa et al., 2009; Namai et al., 2009; Vijayalakshmi et al., 2015). The high NUE/N-insensitive genotypes (NIS-top grain yielders) are defined as genotypes that give more or equal GY with minimal application of N fertilizer when compared to recommended or standard N fertilizer conditions (Hawkesford, 2017). The understanding of GY and its associated NUE traits performance is still largely lagging in the pearl millet diversity panel. However, few studies had explored genotypic variations for NUE at different N levels. In one such report, 20 diverse pearl millet genotypes and few high-yielding hybrids were screened under field conditions at two N levels (Alagarswamy and Bidinger, 1987). In another study, pearl millet hybrids were grown over 3 years in western and eastern Nebraska at four different N levels to determine the optimum N rate for cultivation and concluded that the NUtE trait was less responsive than the N uptake with increased N levels (Maman et al., 2006).

Nitrogen use efficiency, which is dependent on soil or external N supply, is an output of available N uptake, its efficient utilization and remobilization to grain at end of the season. Several studies revealed that improved NUtE might lead to enhanced NUE under low N conditions, particularly in cereals (Moll et al., 1982; Good et al., 2004). NUtE is defined as the genotype ability to assimilate and remobilize N which ultimately convert into GY. It is an essential physiological parameter that unveiled the positive relationship with GY. Whereas in pearl millet comprehensive studies are required to pinpoint the critical factors underlying NUtE under different N levels. GY is a complex trait controlled by a network of multiple traits and their associations. The GY in pearl millet is a result of many yield components, such as grain number, grain weight, tiller number, and panicle number (Rai et al., 2012; Basava et al., 2019). Hence, uncovering the genetic basis of GY, and other related NUE traits under low and high N conditions is a prerequisite to understand the mechanism and also to identify ideal NUE associated traits for selection. Thus, the current study was aimed to evaluate a diverse set of pearl millet lines for their response to GY and NUE related traits under different N levels and also to determine the suitable NUE traits for selection. This study provides useful information toward uncovering the physiological and genetic basis of GY and its related traits under low N which further facilitates the development of low N stress-resilient pearl millet cultivars.

## Materials and Methods

### Experimental Details

The experiment was conducted at an N-depleted precision field of the International Crops Research Institute for the Semi-Arid Tropics (ICRISAT), Patancheru, Telangana, India. The farm is geographically situated at an altitude of 545 m above mean sea level on 17.53°′ N latitude and 78.27°′ E longitude. The climate of the location is semi-arid with an average rainfall of 898 mm. The minimum and maximum temperatures ranged from 38 to 42°C which was observed during the experimental seasons. A set of 380 diverse pearl millet lines consisting of a world diversity panel, Pearl Millet Inbred Germplasm Association Panel-(PMiGAP) (Sehgal et al., 2015), mapping population parents and checks were screened during three seasons (summer 2017, rainy 2017, and summer 2018) in the same field with minimal fertility and moisture gradient. Across all the three seasons, field experiments were carried out in a split-plot alpha-lattice design with 2 m and three-row plots with two replications under three N treatments [N_0_ (without additional N application), N_50_ (@50 kg ha^–^), and N 100 (@100 kg ha^–^)]. Here N source used was urea (46.4% N). Field area with three different N treatments was considered as the main plot and it was divided into six blocks (two replications for each treatment). Each N level block was divided into 25 sub-blocks, and each sub-block consisted of 16 genotypes. Nitrogen depleted plot was developed and maintained at ICRISAT over past many seasons before the inception of the experiment by withholding N application. Before initiating the experiment, important physical and chemical properties of the soil were measured. Soil samples were collected at 15 cm depth from different places and four corners of the plot; mixed composite was used to determine the soil properties (Supplementary Table 1).

### Crop Management

Crop management including land preparation, sowing/planting, management of water, nutrient, disease, and pest control were taken care of throughout the crop period. Seeds were sown on the raised furrows by using a four cone planter by maintaining sufficient spacing of 60 cm from row to row and 15 cm from plant to plant. The crop was fertilized by manual broadcasting as per the treatment. N fertilization was done as per the treatments using urea (46.5%) in two equal splits at 20–25 and 40–45 days after sowing. Besides, other nutrients such as single super phosphate @250 kg ha^–1^ and muriate of potash (60% K_2_O) @50 kg ha^–1^ was also applied at the time of sowing. All the pearl millet genotypes were evaluated for a total of 11 traits across three consecutive growing seasons under different N regimes. The measured phenotypic traits were (1) SPAD chlorophyll content (soil plant analytical device), (2) leaf number (LN), (3) LA, (4) GY, (5) dry stover yield (DSY), (6) N percent in dry stover (NPS), (7) N uptake in grain (NUpG), (8) N uptake in stover (NUpS), (9) total nitrogen uptake (TNUp), (10) NUtE, and (11) nitrogen harvest index (NHI). The phenotypic data were averaged across three seasons in each genotype for the first five traits SPAD chlorophyll content, LN, LA, GY, and DSY. For the remaining six NUE related traits data was presented as a pooled mean of two summer seasons, due to lack of quality data in the rainy season.

### Physiological Traits

#### SPAD Chlorophyll Content (Soil Plant Analytical Device)

Soil plant analytical device chlorophyll content was measured by using Minolta Corporation’s Chlorophyll SPAD-502 plus, United States. Plants from all the treatments were marked a day before with different color ribbons. Fully expanded uppermost leaves at 45 days’ stage (from the day of leaf emergence) were selected and an average of three reads was recorded from a total of three plants from the middle row.

#### Leaf Area and Leaf Number

In order to examine the LA and LN, leaf samples were collected approximately 80 days after emergence for all the treatments and replications. Healthy plant per plot was harvested from the middle row in the field in early morning and on the same day, specific LA and number were measured by passing in all the collected leaves of the plant through LA meter LI-3100C (LI-COR, United States).

#### Grain and Stover Yield

At the crop harvesting stage, GY was measured after threshing sundried panicles to remove the grains from their central rachis. Harvested plants were selected from the middle row and expressed in grams (g) per 2-meter area. Similarly, DSY was also measured after drying the stover samples of all the middle row plants without panicles and expressed in grams per 2-meter area.

### Nitrogen Estimation and Nitrogen Indices

#### Nitrogen Estimation

Nitrogen content in grain and stover samples was estimated by using the sulfuric acid-selenium digestion method. Grain and stover were made into fine powder by using clone mixture (*cyclone sample mill*) and 0.250 gm was used for nitrogen estimation in the Charles Renard Analytical Laboratory at ICRISAT, Patancheru. Pre-weighed samples were digested with the sulfuric acid-selenium and then analyzed (Sahrawat et al., 2002) by using an auto-analyzer (Skalar SAN System, AA Breda, Netherlands). Nitrogen concentration is expressed as Nitrogen% (N%).

#### Different Nitrogen Use Efficiencies

Total N uptake was calculated from the sum of grain uptake and stover N uptake values. Then different N efficiencies were calculated as per the formulas given by Fageria et al. (2010).

##### Nitrogen utilization efficiency (NUtE)

NUtE (kg grain kg^–1^ N) = GY/TNUp.

##### Nitrogen harvest index (NHI)

NHI (%) = (NUpG/TNUp) × 100.

### Statistical Analysis

The phenotypic mean values over the replications of each genotype for all the traits were prepared and the best linear unbiased estimates (BLUE) for genotypes were estimated at each and across N levels using Genstat software, 20th edition. The minimum, maximum, and BLUE of each trait at each N level were calculated. ANOVA was performed for the individual and pooled seasons by modeling individual season’s residuals variance using Genstat software, 20th edition (VSN International, 2019). Fisher’s *t*-test was used to ascertain the significant difference among the genotypes, treatments and interactions. Pearson’s correlations coefficient analysis was performed by using pooled data of two summer seasons for all the 11 traits to identify the relationship between traits at each level of N treatments.

### Cluster Analysis

Hierarchical cluster analysis was carried out using the Euclidean distance metric and UPGMA method (un-weighted paired group method and arithmetic averages)^1^. By using the pooled data of three seasons treatment wise (N_0_, N_50_, and N_100_) cluster analysis was performed for the five traits viz., SPAD chlorophyll content, LN, LA, GY, and DSY.

## Results

According to the fertilizer map, the available nitrogen (N) content of the soil sample was divided in to low, medium, and high. Therefore, low N conditions were observed when soil nitrogen content is below 280 kg/ha (critical limits of the soil maintained as per ICRISAT guide limits). The selected experimental field was not used for any cultivation from the past 5–6 years and before starting the trial, soil samples were collected across the field and undergone soil nutrient analysis. Nutrient analysis (Supplementary Table 1) revealed 0.5% organic carbon (OC), indicating a low available N in the soil and the basal N availability was common across all the N regimes before the initiation of the experiment. In addition, the soil micronutrient availability was in the medium range. The basal N availability in the soil is common for all the treatments but coming to the N_50_ and N_100_ regimes, an additional N source in the form of urea was applied as per the protocol mentioned in the methodology section. Overall, the N_0_ regime was maintained strictly without any additional supply of nitrogen.

A total of 380 pearl millet genotypes including the diversity panel, parents of mapping populations, and checks were screened for 11 physio-agronomic and NUE traits at three nitrogen levels (0, 50, and 100% of the recommended N doses) with two replications. Data recorded were averaged across the three seasons for each trait in each N level. Results revealed the native variation across the genotypes toward N response which had given the scope to identify nitrogen use efficient lines under low and high N conditions for the marginal and favorable ecologies, respectively. ANOVA results revealed significant variations among the genotypes, treatments and their interactions (season × treatment (N levels), season × genotype, treatment × genotypes, and season × treatment × genotype). The pooled means over three seasons in each genotype in each N level for five physio agronomic traits were provided in Supplementary Table 2. The pooled mean of over two seasons for six N related traits in each genotypes were provided in Supplementary Table 3.

### Physiological Traits

Key physiological traits viz., SPAD chlorophyll content, LN, and LA were recorded for all three seasons. Significant variations across the genotypes under different N regimes were noticed. Under N_0_, SPAD chlorophyll content was ranged from 28.18 to 49.11 with an average of 37.85. Whereas in N_50_, it was ranged from 32.16 to 50.1 with an average of 40.59. In N_100_, the values were ranged from 29.04 to 49.07 with an average of 41.77. In N_0_, SPAD chlorophyll content values were reduced by 6.8 and 9.4% compared to N_50_ and N_100_ conditions, respectively. LN was decreased under N_0_ conditions by 19.3 to 29.6% compared to N_50_ and N_100_ conditions, respectively. The range of LA was lower under N_0_ compared to N_100_ conditions. The mean of LA was reduced significantly under the N_0_ condition compared to the N_100_ condition. In addition, the analysis of variance (ANOVA) results revealed that the effect of genotype, treatments and their interactions were significant (*P* < 0.001) for all the three physiological traits studied. The data was averaged across the three seasons for each trait in each N level. Descriptive statistics over the three seasons, and ANOVA results for all the physiological traits were provided in Table 1.

**TABLE 1:** Summary statistics and ANOVA results from three seasons pooled data for physiological and agronomic traits under three N levels.

Traits		SPAD chlorophyll content	LN	LA	GY (g/2m^2^)	DSY g/2m^2^)
Range	N_0_	37.85	17.89	722.59	67.04	202.06
	N_50_	40.59	22.18	1212.4	92.61	246.16
	N_100_	41.77	25.40	1486.04	88.89	264.41
Mean	N_0_	28.18–49.11	8.47–34.81	202.64–2220	9.64–159.71	58.47–806.09
	N_50_	32.16–50.1	13.25–42.77	652.35–2967.8	20.72–236.43	62.42–0.740.78
	N_100_	29.04–49.07	10.30–41.73	363.65–2742.06	20.48–242.12	73.92–678.87
Percent reduction	N_0_ compared with N_50_	6.8	19.3	40.4	27.6	17.9
	N_0_ compared with N_100_	9.4	29.6	51.4	24.6	23.6
	N_50_ compared with N_100_	2.8	12.7	18.4	–4.2	6.9
F statistic	S	12.45***	345.29***	742.27***	580.24***	609.32***
	T	2.27^ns^	40.48***	227.09***	213.16***	55.08***
	S × T	0.67^ns^	15.21***	67.86***	67.77***	93.49***
	G	5.94***	7.44***	8.48***	9.48***	14.97***
	S × G	4.65***	5.19***	7.77***	6.87***	7.54***
	T × G	2.49***	3.5***	3.6***	2.86***	4.01***
	S × T × G	2.17***	2.87***	4.3***	2.32***	2.73***

SPAD, Chlorophyll content; LN, leaf number; LA, Leaf area; GY, grain yield; DSY, dry stover yield; G, Genotype; S, Season; T, treatment. F statistic values with asterisk indicate significant at levels of 0.001 (***) and NS denote for non-significant.

### Agronomic Traits

#### Grain Yield

In N_0_, the average GY of the tested genotypes was 67.04 g and ranged from 9.64 g (IP13363) to 138.48 g (IP18621). Similarly, significant genotypic variations were observed in other treatments. In N_50_, GY values varied from 20.72 g (Tift238 D1) to 168.85 g (IP16096) with a mean of 92.61 g. Whereas in N_100_, GY ranged from 20.48 g (IP13363) to 164.87 g (ICMV-IS89305) with a mean of 88.69 g. Nitrogen in limited condition (N_0_) resulted in a 27.6 and 24.6% reduction in GY compared to N_50_, and N_100_, respectively. The ANOVA results indicated that the effect of genotype, treatments were significant (*P* < 0.001) and their interactions were also significant between seasons × treatment (*P* < 0.001), seasons × genotypes (*P* < 0.001), treatment × genotype (*P* < 0.001), and season × treatments × genotype.

**FIGURE 1:**
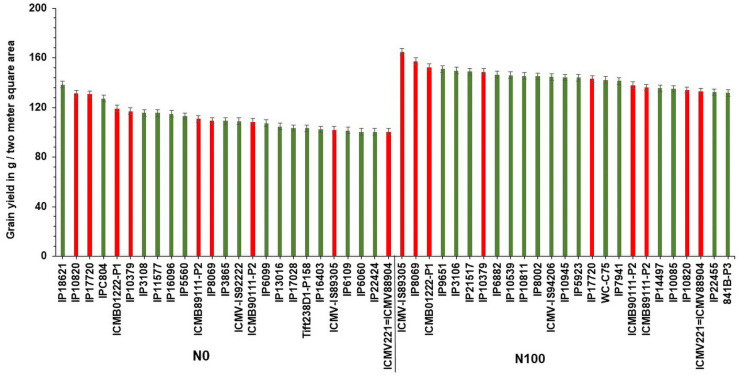
Top 25 high grain yielding genotypes under N_0_ and N_100_ conditions. Total 9 genotypes (in red color) viz. IP 10820, IP 17720, ICMB 01222-P1, IP 10379, ICMB 89111-P2, IP 8069, ICMB 90111-P2, ICMV IS89305, and ICMV 221 exhibited high grain yield in both N_0_ and N_100_ conditions.

**FIGURE 2:**
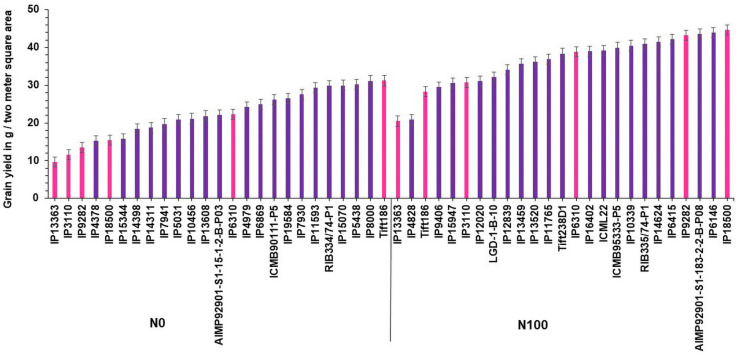
Top 25 poor yielding genotypes for grain yield under N_0_ and N_100_ conditions.

Based on GY data, top 25 (N-insensitive (NIS-Top grain yielders) and least 25 (N-sensitive (NS-Poor grain yielders) genotypes were identified under N_0_ conditions (Table 2). Out of 25 NIS lines, nine genotypes (IP10820, IP17720, ICMB01222-P1, IP10379, ICMB89111-P2, IP8069, ICMB90111-P2, ICMV-IS89305, and ICMV221) were common in the top 25 lines at the N_100_ level which shows the genotype plasticity toward N_0_ and N_100_ conditions (Figure 1). Similarly, the top 25 high yielding genotypes were identified in N_100_. The 25 least grain yielding genotypes [N-sensitive (NS)] in N_0_ and N_100_ conditions were presented in Figure 2.

**TABLE 2:** Top 25 NIS and NS lines under low nitrogen (N_0_) with contrasting NUE grain yield derived from the pooled mean over three seasons.

S. No	Genotypes	NIS	Genotypes	NS
1	IP18621	138.42	IP13363	9.64
2	IP10820	131.19	IP3110	11.54
3	IP17720	130.81	IP9282	13.56
4	IPC804	127.12	IP4378	15.27
5	ICMB01222-P1	119.13	IP18500	15.43
6	IP10379	116.92	IP15344	15.75
7	IP3108	115.54	IP14398	18.45
8	IP11577	115.52	IP14311	18.72
9	IP16096	114.84	IP7941	19.73
10	IP5560	113.03	IP5031	20.88
11	ICMB89111-P2	110.84	IP10456	21.15
12	IP8069	109.29	IP13608	21.84
13	IP3865	109.15	AIMP92901-S1-15-1-2-B-P03	22.09
14	ICMV-IS92222	108.9	IP6310	22.31
15	ICMB90111-P2	108.34	IP4979	24.2
16	IP6099	107.22	IP6869	24.92
17	IP13016	104.61	ICMB90111-P5	26.13
18	IP17028	103.4	IP19584	26.53
19	Tift238D1-P158	103.31	IP7930	27.56
20	IP16403	102.3	IP11593	29.33
21	ICMV-IS89305	101.87	RIB334/74-P1	29.81
22	IP6109	101.32	IP15070	29.94
23	IP6060	100.37	IP5438	30.23
24	IP22424	100.3	IP8000	31.19
25	ICMV221 = ICMV88904	100.22	Tift186	31.22

Furthermore, agronomic data was provided for the top five lines *viz*., IP 18621, IP 10820, IP 17720, IPC 804, and ICMB01222-P1, out of selected 25 NIS lines (Supplementary Table 4). Interestingly, days to 50% flowering (DF50%) and plant height (PH) was more in all the tested genotypes at N_0_, indicating the genotype efficiency in terms of agronomic traits. These genotypes can be used as parents in the pearl millet breeding program to develop N efficient genotypes under low N input conditions.

#### Dry Stover Yield

In N_0_, DSY ranged from 58.47 g (LGD-1-B-10) to 806.19 g (IP8863) with a mean of 256.3 g. Whereas in N_50_, values ranged from 62.42 g (IP13363) to 740.78 g (IP11584) with a mean of 246.16 g. In N_100_, the average DW of 380 genotypes was 264.41 g, and values varied from 73.92 g (Tift23D2B1-P1-P2) to 678.87 g (IP8786). Application of N significantly increased DSY by 12.6% in N_50_ and 18.02% in N_100_ compared to the N_0_ condition. Significant differences were observed for dry stover weight among the genotypes (*P* < 0.001), and interactions were also significant between seasons × treatment (*P* < 0.00), seasons × genotypes (*P* < 0.001), treatment × genotype (*P* < 0.001), and season × treatments × genotype.

**FIGURE 3:**
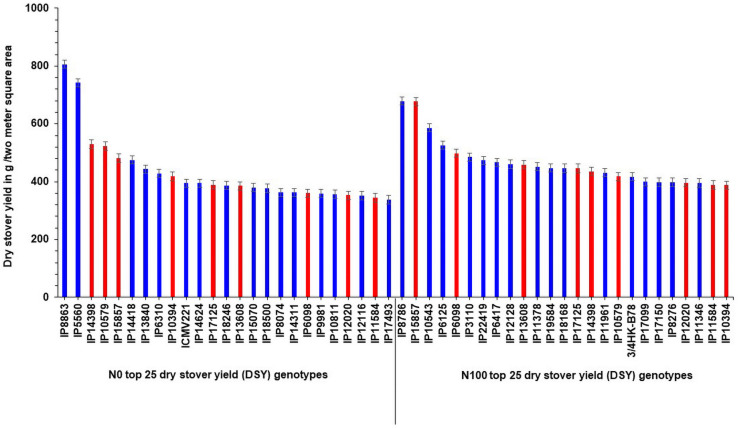
Top 25 high dry stover yielding (DSY) genotypes under N_0_ and N_100_ conditions. Total 9 genotypes (highlighted in red color) viz., IP 14398, IP 10579, IP 15857, IP 10394, IP 17125, IP 13608, IP 6098, IP 12020, and IP 11584 exhibited high dry stover yield (DSY) in both N_0_ and N_100_ conditions.

**FIGURE 4:**
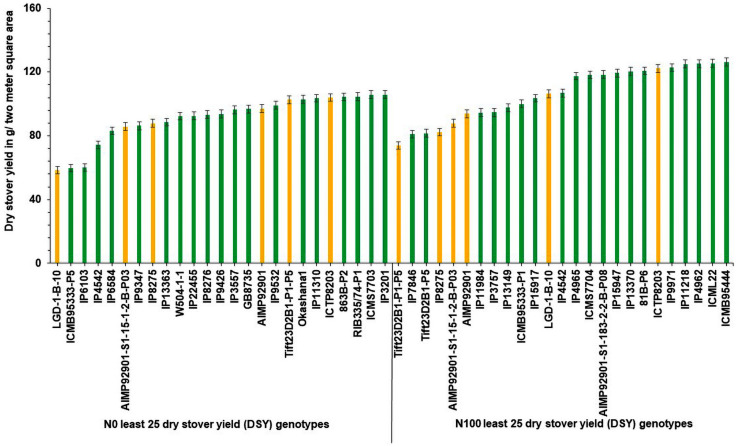
Least performing genotypes for dry stover yield (DSY) under N_0_ and N_100_ conditions.

Interestingly, out of the top 25 high dry stover yielding lines, nine genotypes (IP14398, IP10579, IP15857, IP10394, IP17125, IP13608, IP6098, IP12020, and IP11584) were common in the top 25 lines at N_100_ condition (Figure 3). At both N levels, the top 25 poor-performing dry stover genotypes were also identified (Figure 4).

In addition, three high yielding dual purpose genotypes (IP5560, IP15857, and IP17125) were identified in the top 25 GY and DSY genotypes under N_0_. Whereas in N_100_, only one common high yielding genotype (IP3106) was identified. Descriptive statistics over the three seasons, and ANOVA results for the agronomic traits were provided in Table 1.

### N Content in Grain and Stover

Nitrogen percent in grain (NPG) varied from 1.18% (IC804) to 2.43% (Tift 186), with an average of 1.61% in N_0_ condition. Whereas in N_50_, NPG ranged from 1.19% (9444) to 2.43% (IP4942) with a mean of 1.67%. Under N limiting conditions NPG was reduced by 3.6 and 11.4% as compared with N_50_ and N_100_ conditions, respectively. In N_50_, an average mean of NPG decreased by 8.24% as compared to N_100_. The response of genotypes in the N_100_ condition varied significantly from 1.27% (IP10539) to 2.59% (IP4828) with a mean of 1.82%. Significant differences were observed in NPG among the genotypes (*P* < 0.001), treatments (*P* < 0.001), and interactions and are also significant between seasons × treatment (*P* < 0.00), seasons × genotypes (*P* < 0.001), and season × treatments × genotype except for treatment × genotype (0.677).

Genotypic variations found in NPS in N_0_ and the minimum and maximum value of NPS ranged from 0.42% (IP8786) to 1.08% (ICMB90111-P5) with a mean of 0.61%. The average NPS was more in N_0_ compared to N_50_ and N_100_ conditions. Significant variations were observed in all the interactions except season × treatment (0.6771) and treatment × genotypes (0.0102).

### Nitrogen Uptake for Dry Stover

Genotypic variations were observed for NUpS across the N levels. The mean of all genotypes was 1.65 kgha^–1^ ranged from 3.34 kgha^–1^ (ICMB9533-P5) to 8.03 kgha^–1^ (IP5560) and decreased by 9.8 and 14.45% in N_0_ compared to N_50_ and N_100_, respectively. Whereas, in N_50_, the values varied from 0.51 kgha^–1^ (AIMP92901-S1-15-1-B-P3) to 8.03 kgha^–1^ (IP10543) with a mean of 1.83 kgha^–1^ and decreased by 5.67% when compared with N_100_. The minimum and maximum values of NUpS varied from 0.38 kgha^–1^ (IP155363) to 1.08 kgha^–1^ (IP11584) with a mean of 6.59 kgha^–1^ under N_100_ condition.

### Total Nitrogen Uptake

Application of N fertilizer resulted in an increase of TNUp and also revealed significant genotypic variations among the genotypes with in the treatments. Under N_0_, the values ranged from 1.09 to 10.47 with an average of 3.03 kg ha^–1^ and decreased by 14.4 and 21% as compared with N_50_ and N_100_, respectively. Whereas in N_50_ and N_100_, values varied from 1.08 (IP11229) to 9.57 (843B) and 0.58 (IP13363) to 8.97 (IP11584) with a mean of 3.54 and 3.83 kg ha^–1^, respectively. The results of ANOVA for TNUp indicated significant effects for all the interactions except treatments.

### Nitrogen Utilization Efficiency

Large variation was observed for NUtE of genotypes, ranged from 2.40 (IP3110) to 50.60 (IP3557) with an average of 30.81 under N_0_ and the efficiency was reduced as compared with N_50_ and N_100_ by 7.3 and 5.95%, respectively. Whereas in N_50_, values varied from 10.63 to 50.86 with a mean of 33.22, and a 7.57% reduction was observed as compared with the N_100_ condition. The application of N fertilizer (N_100_) resulted in a significant increase in NUtE. The minimum and maximum NUtE were observed in IP31110 (10.06) and IP10456 (56.25), respectively with an average of 32.76. Significant differences were observed among the genotypes (*P* < 0.001), treatments (*P* < 0.001), and seasons × treatments (*P* < 0.001), seasons × genotypes (*P* < 0.001), treatment × genotypes and interactions were also significant between season × treatments × genotype.

**FIGURE 5:**
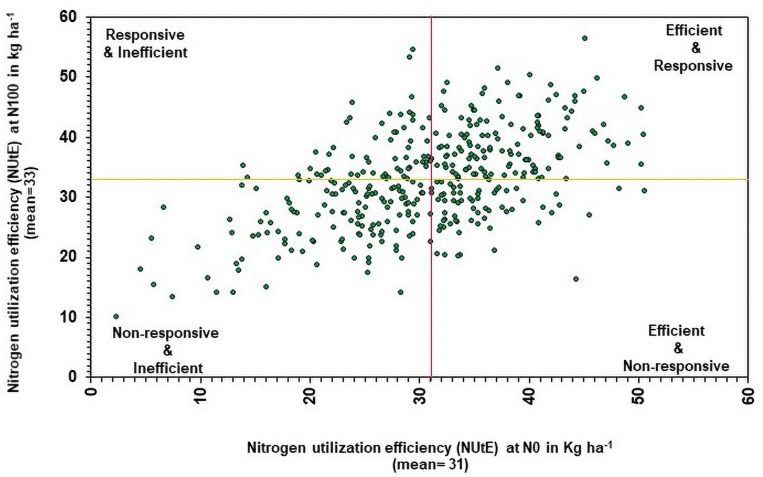
Relationship between genotypes performance of nitrogen utilization efficiency (NUtE) under low (N_0_) and recommended N (N_100_) conditions. The yellow line represents the mean of NUtE at N_100_ and the purple line represents the mean of NUtE at N_0_ (pooled data from two summer seasons 2017 and 2018).

Nitrogen utilization efficiency under N_0_ was compared with NUtE under N_100_ to determine the genotypic efficiency and their responsiveness to N. Based on the NUtE, the genotypes were classified into four groups (Figure 5) viz., (1) N efficient non-responsive (NENR), (2) N efficient responsive (NER), (3) N responsive inefficient, and (4) N inefficient non-responsive (Rengel and Graham, 1995; Worku et al., 2007). In this study, NutE data recorded in 374 genotypes across different N levels. The average NUtE of 374 genotypes under N_0_ (30.81) and N_100_ condition (32.76) were considered as cut off for the identification of genotypic efficiency and responsiveness for N use. Under low N, above-average genotypes were considered as efficient and below-average genotypes were considered as inefficient. Similarly, in N_100_, above-average genotypes were considered as responders, and below-average genotypes are considered as non-responders. Overall, efficient genotypes are higher in the utilization of absorbed N over inefficient genotypes.

### Nitrogen Harvest Index (NHI)

The nitrogen harvest index varied from 5.46% (IP3110) to 74.52% (ICMB 95333-P5) with an average of 47.56% in N_0_ condition and a reduction of about 10.3 and 15.43% compared to N_50_ and N_100_ condition, respectively. Whereas in N_50_, NHI values ranged from 21.10% (IP10488) to 84.02% (IP5923) with a mean of 53.0%. The response of genotypes under the N_100_ condition varied significantly and ranged from 23.51% (IP12020) to 78.89% (IP6110) with a mean of 56.24%. Further, the NHI reduction under N_0_ was about 10.3 and 15.43% as compared with N_50_ and N_100_, respectively. In N_50_, a reduction of NHI about 5.46% was observed compared to the N_100_ condition. Significant differences were observed among the genotypes (*P* < 0.001), treatments (*P* < 0.001), and interactions were also significant between seasons × treatment (*P* < 0.001), seasons × genotypes (*P* < 0.001), and season × treatments × genotype except for the treatment × genotype (0.677) (Table 3).

**TABLE 3:** ANOVA for N-related traits under three nitrogen levels during Summer 2017 and 2018.

Traits		NPG (%)	NPS (%)	NUpS (kg ha^–1^)	TNUp (kg ha^–1^)	NUtE (kg ha^–1^)	NHI (%)
Range	N_0_	1.18–2.43	0.42–1.08	0.34–8.03	1.09–10.47	2.42–50.6	5.46–74.52
	N_50_	1.19–2.43	0.4–0.94	0.51–6.27	1.08–9.57	10.63–56.86	21.07–84.02
	N_100_	1.27–2.59	0.32–1.03	0.38–6.59	0.58–8.97	10.06–56.27	23.51–78.89
Mean	N_0_	1.61	0.61	1.65	3.03	30.81	47.56
	N_50_	1.67	0.56	1.83	3.54	33.22	53.00
	N_100_	1.82	0.56	1.94	3.83	32.76	56.24
Percent reduction	N_0_ compared with N_50_	3.6	–8.9	9.8	14.4	7.3	10.3
	N_0_ compared with N_100_	11.54	–8.93	14.95	20.89	5.95	15.43
	N_50_ compared with N_100_	8.24	0.00	5.67	7.57	–1.40	5.76
F statistic	S	1808.86***	717.71***	7105.54***	12241.28***	129.2***	45.79
	T	39.22***	12.04***	1.75^ns^	2.52^ns^	43.13***	43.8***
	S × T	0.39^ns^	5.39**	148.57***	212.2***	172.45***	16.36***
	G	9.34***	3.48***	7.7***	6.66***	9.86***	8.33***
	S × G	5.19***	2.67***	6.44***	4.58***	4.5***	4.72***
	T × G	1.85***	1.15**	3.91***	3.07***	2.09***	2.47***
	S × T × G	1.47***	1.1^ns^	2.26***	1.85***	2.09***	2.31***

*NPG. N percent in grain; NPS, N percent in dry stover; NUpS, N uptake in stover; TNUp, total nitrogen uptake; NUtE, nitrogen utilization efficiency; NHI, nitrogen harvest index; G, Genotype; S, Season; T, treatment.*

F statistic values with asterisk indicate significance at the level of 0.01 (**) and 0.001 (***) and NS denotes no significance.

### Phenotypic Correlation

**FIGURE 6:**
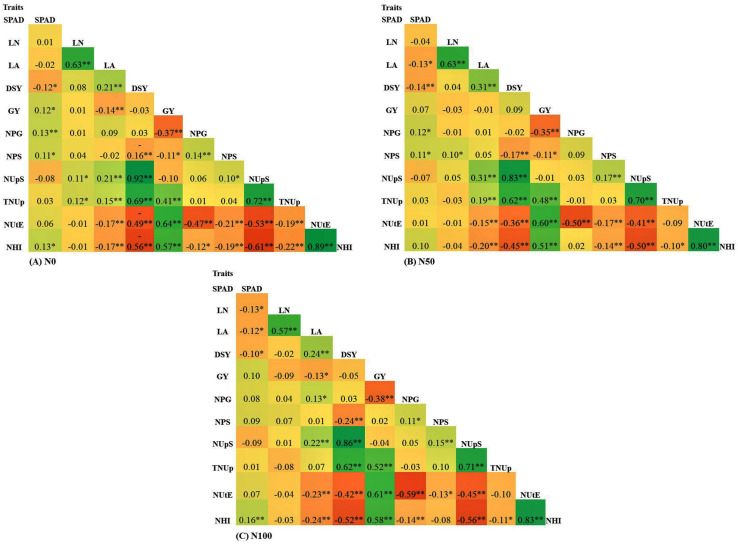
Pearson correlation for traits underlying NUE in 380 global diverse pearl millet genotypes (PMiGAP) under three different nitrogen (N) regimes carried out in 2017/18 (data pooled from two summer seasons). **(A)** Correlations among eight traits at N_0_ condition (N_0_). **(B)** Correlations among eight traits at N_50_ condition. **(C)** Correlations among eight traits at N_100_ condition. ^∗^ indicates < 0.05 significance, ^∗∗^ indicates < 0.01 significance.

Correlation coefficient analysis was performed to identify the interrelation among the traits for all the N levels. The complete list of correlation coefficient values among the traits in each N level is provided in Figure 6. GY showed a significant positive correlation with TNUp, NUtE, and NHI. The results revealed that the GY of pearl millet was increased through the selection of higher NUtE and NHI lines. Furthermore, GY negatively correlated with DSY, NPS, NPG, and NUpS at both N_0_ and N_100_ conditions. The results suggested that the higher GY was, the lower DSY, NPS, NPG, and NUpS. Further DSY showed a negative significant correlation with all the measured traits except NUpS and TNUp across N levels. Interestingly NUtE showed a positive correlation with GY and NHI across N levels. SPAD chlorophyll content was found to be positively correlated with all traits except LA, DSY, and NUpS across all the treatments. Further LA was negatively correlated with GY, NUtE, and NHI across the treatments.

### Cluster Analysis

**FIGURE 7:**
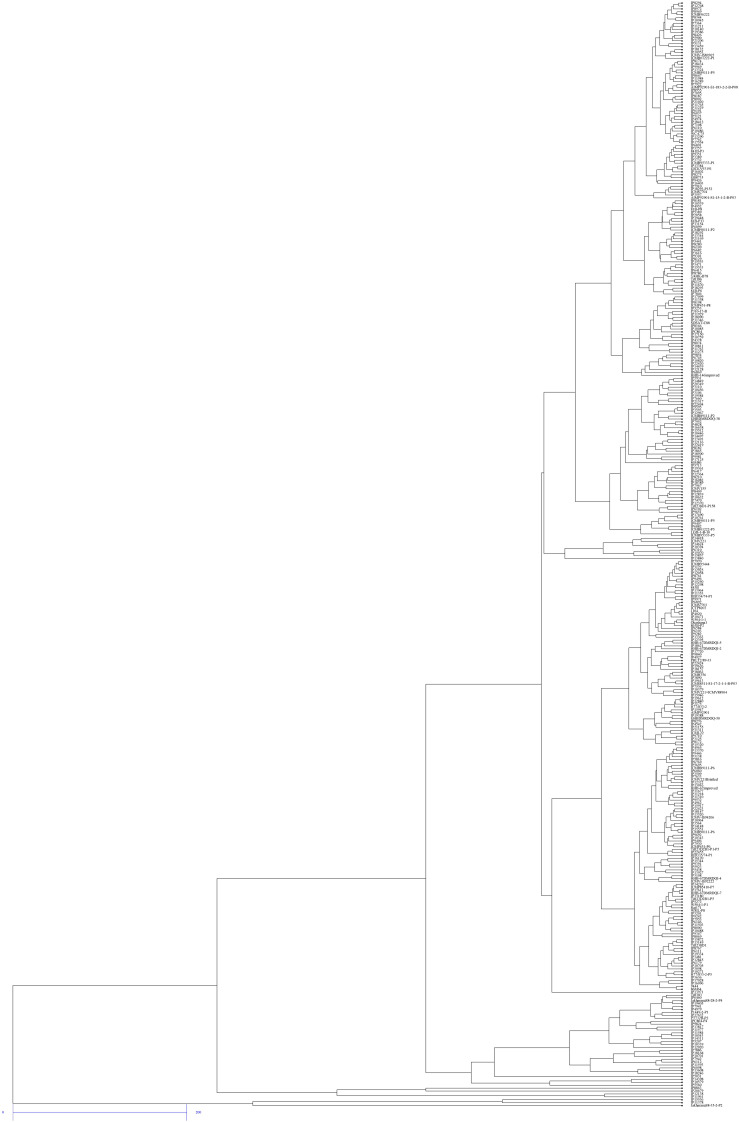
Dendrogram of 380 pearl millet genotypes for five traits under low nitrogen conditions (N_0_) by the UPGMA method.

**FIGURE 8:**
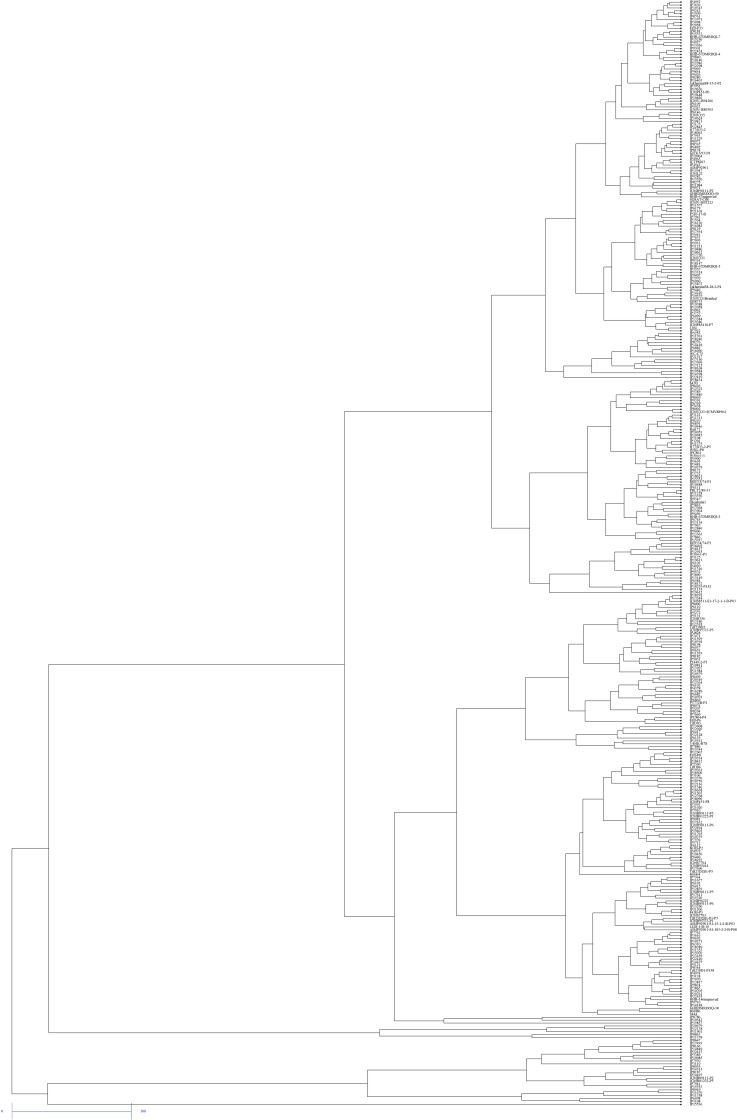
Dendrogram of 380 pearl millet genotypes for five traits under recommended conditions of nitrogen (N_100_) by the UPGMA method.

Hierarchical cluster analysis of 380 pearl millet genotypes revealed large genotypic variation in five traits viz., SPAD chlorophyll content, LN LA, GY, and DSY. This analysis is useful for grouping genotypes for NUE. Therefore, clustering 380 genotypes based on five traits identified two to three major clusters. Based on this, genotypes were classified into high, medium, and low-performing genotypes. At both N levels, clusters I and II contained better-performing genotypes and also found the least dissimilarity among the genotypes. Interestingly few common genotypes viz., IP 15536, IP 6098, IP11961, IP8863, IP11378, IP 20679, and IP 12138 were identified in N_0_ and N_100_ levels in the first two clusters, implying that genotype plasticity toward N under N_0_ and N_100_ conditions (Figures 7, 8). Cluster III contained more number of medium performing genotypes that form a maximum number of sub clusters at both N levels. Finally, the least performing genotypes were present in Cluster IV. Overall, the maximum dissimilarity (from all these matrices) was observed between cluster I and IV.

## Discussion

Nitrogen (N) is an essential macronutrient required for plant growth and is often a limiting factor for crop yield. From the past 50 years of agriculture, augmented food production was attained by the extensive application of N fertilizer in combination with N-responsive cultivars across the globe. Excessive application of N fertilizer is becoming expensive which accounts for the loss of economic profit to the farmers along with negative impacts on the environment (Raun et al., 2002; Hawkesford and Griffiths, 2019). Hence to overcome this problem, a clear understanding of genotype behavior, identification and development of genotypes (without compromising GY) with high NUE under low N conditions is a paramount need for improving NUE.

Therefore, the present study aimed to identify the N-Sensitive (NS) and N-insensitive (NIS) genotypes and also to determine the traits regulating low N tolerance. However, accurate and reliable phenotyping under low N input is challenging and influenced by the genotype (G), environment (E), and G × E interactions (Chen et al., 2014; Rao et al., 2018). Moreover, there are limited studies available in pearl millet toward the identification of nitrogen insensitive genotypes under low N conditions at field level. Based on the current knowledge, this study presents the first report of genetic variations for NUE in the global association panel, Pearl Millet inbred Germplasm Association Panel (PMiGAP) (PMiGAP collected from a large set of diverse germplasm and breeding lines) and from mapping population parents. Notably, very few pearl millet breeding programs are targeting the development of low N tolerance traits which are a must for sustainable agriculture with minimal negative impacts on the environment. Components of NUE were studied in pearl millet hybrids at two N levels found that the efficient genotype (Souna B) had a 32% of higher NUE value than the N inefficient Indian genotype (BJ104) (Alagarswamy and Bidinger, 1987). Another study revealed that NUtE contributes more to genetic variation in NUE (Maman et al., 2006).

In this study, a total of 380 diverse pearl millet genotypes were characterized for SPAD chlorophyll content, LN, LA, GY, DSY, and NUE related traits at three different N levels under field conditions. Notably, diverse responses have been observed among the genotypes across N levels, despite a similar growth conditions and an equal amount of N fertilizer in a given N level. These observed genotypic variations purely reveal the genotype plasticity toward traits. This set of lines used in this study which are collected from different regions across the globe and these lines were used for various abiotic stress studies to identify the tolerant genotypes. Previous studies in rice, maize, wheat, and oilseed rape, etc., have established significant genetic variation for NUE-related traits with large germplasm panels, hybrids, open-pollinated and recombinant inbred line populations (Chen et al., 2014; Li et al., 2015; Vijayalakshmi et al., 2015; Ertiro et al., 2017; He et al., 2017; Rao et al., 2018). Generally, the results of ANOVA explained statistical differences among the treatments and genotypes and their interactions. In the current study, ANOVA results revealed significant variations among the genotypes and treatments for all measured traits except a few traits (Tables 1, 3). These results were indicating that the environment under different N levels was a crucial factor in explaining the genotypic variance of GY and its related traits, which are strongly influenced by the relative contribution of G × E interactions under field evaluation. The results concur with other reported field experiments in rice (Srikanth et al., 2016) and wheat (Sial et al., 2005; Belete et al., 2018).

In the present study, SPAD chlorophyll content significantly increased with nitrogen application, which might be sufficient availability of N in the leaf. Similar results of increased SPAD chlorophyll content by increasing N application was reported by Kitajima and Hogan (2003), Pramanik and Bera (2013). The relation between the slow or controlled release of N fertilizers supporting more N absorption and related physiological mechanisms has been well studied in different crops (Li et al., 2003; Long et al., 2013). LA of all the pearl millet genotypes was significantly increased with N application which might be attributed to translocation of N to leaves, which brings variation in plant architecture and leaf internal structure (Singh et al., 2004; Mhaskar et al., 2005). Reduction in LN under N_0_ condition was observed, due to deprivation of N, thereby reduced source size and hindering the plant development.

Across the year on average, GY ranged from 9.64 g to 159.71 g (N_0_), 20.72 g to 236.43 g (N_50_), and 20.48 g to 242.12 g (N_100_), indicating the genotypic variability in GY at different N regimes. In general, GY increased was correlated with the N application rate, which might be due to sufficient nitrogen availability. Recently similar kind of results was reported in a diverse set of foxtail millet genotypes (Bandyopadhyay et al., 2020). In wheat, GY and quality traits were evaluated by Šarčević et al. (2014) at low and normal N and found that GY was reduced by 10% under low N condition compared to normal condition. The current results also are in tune with the earlier reports as there was an increased GY with increase N levels. Hawkesford and Griffiths (2019) reported that split application of N is the best option to recover the maximum applied N in the form of harvested grain/increased GY and found a strong significant relationship between GY and physiological traits.

Remarkably, 25 high yielding genotypes were identified under the N_0_ condition and considered as N-insensitive (NIS). Out of 25, nine genotypes (IP10820, IP17720, ICMB01222-P1, IP10379, ICMB89111-P2, IP8069, ICMB90111-P2, ICMV-IS89305, and ICMV221 = ICMV88904) are common in the top performing 25 lines under N_100_ condition, which shows the genotype plasticity toward varying N levels in these genotypes. These selected genotypes are good genetic resources to breed for tolerance to low N conditions. Top 25 high yielding genotypes in N_100_ can be considered to have the best acceptances for cultivation where soils are fertile and when followed the ideal N levels for cultivation. These identified genotypes could be used to improve GY, along with higher NUE. Numerous studies have reported utilization of N efficient lines with enhanced GY in the farmer fields which may help to reduce fertilizer input as well as increase the profitability of farm operations (Würschum, 2012; Vijayalakshmi et al., 2015; He et al., 2017). Likewise, selected NIS lines with high NUE will certainly play a role in reducing environmental pollution and could increase economic profit to farmers.

Pearl millet is an ideal fodder crop for feeding livestock and has unique features like dry season crop, production environments having low nitrogen, high photosynthetic efficiency, and high dry forage capacity (Govintharaj et al., 2018). Several reports depict the dry fodder demand and it would require approximately 568 million tonnes across the globe by the year 2030 (Govintharaj et al., 2018). To tackle this problem, high fodder yielding genotypes need to be identified. Generally, most of the genotypes have lower dry matter production under low N conditions, hence identification of genotypes with high dry matter production under low N conditions is important for sustainable fodder production. In this framework, the top and least performing 25 genotypes for DSY were identified under N_0_ and N_100_ conditions (Table 4).

**TABLE 4:** Dry stover yield (DSY) for top and least 25 genotypes under N_0_ condition (derived from pooled data of three seasons).

S. No	Genotypes	Top 25 DSY	Genotypes	Least 25 DSY
1	IP8863	806.09	LGD-1-B-10	58.47
2	IP5560	741.82	ICMB95333-P5	59.72
3	IP14398	529.71	IP6103	60.13
4	IP10579	523.27	IP4542	74.33
5	IP15857	481.52	IP6584	83.17
6	IP14418	475.12	AIMP92901-S1-15-1-2-B-P03	85.84
7	IP13840	443.79	IP9347	86.42
8	IP6310	428.9	IP8275	87.84
9	IP10394	419.14	IP13363	88.54
10	ICMV221	395.32	W504-1-1	92.31
11	IP14624	395.1	IP22455	92.39
12	IP17125	389.29	IP8276	93.25
13	IP18246	387.36	IP9426	93.73
14	IP13608	386.07	IP3557	96.39
15	IP15070	380.06	GB8735	96.83
16	IP18500	377.18	AIMP92901	97.12
17	IP8074	362.87	IP9532	99.22
18	IP14311	362.63	Tift23D2B1-P1-P5	102.62
19	IP6098	360.08	Okashana1	102.92
20	IP9981	359.06	IP11310	103.67
21	IP10811	356.19	ICTP8203	104
22	IP12020	353.03	863B-P2	104.48
23	IP12116	351.76	RIB335/74-P1	104.52
24	IP11584	344.58	ICMS7703	105.77
25	IP17493	337.88	IP3201	105.86

These contrasting genotypes can be further exploited in the breeding program and molecular studies to develop varieties with high NUE for biomass. Irrespective of N, the common nine genotypes (IP14398, IP10579, IP15857, IP10394, IP17125, IP13608, IP6098, IP12020, and IP11584) were identified and can produce more dry matter production under both N_0_ and N_100_ conditions. These genotypes need further physiological and molecular characterization under low nitrogen conditions to understand the molecular basis for low nitrogen tolerance and biomass production. Overall, observed genetic variability in pearl millet genotypes are suited well for improving DSY, can be utilized in the breeding program to enhance livestock productivity. Studies on various crops to determine the relationship between nitrogen levels and biomass traits have shown that N fertilizer application increased the biomass related traits, including DSY (Chan-Navarrete et al., 2014). The present results were tuned with these observations as DSY was more in N_100_ condition than in N_0_ condition; the increasing quantity was up to the significant levels. Another study revealed wide genetic variations for biomass traits and stover nitrogen in pearl millet germplasm (Gupta et al., 2015). Few studies revealed that significant genetic variation present in pearl millet for biomass traits in dual-purpose hybrids and open-pollinated varieties (OPVs), populations, and top cross hybrids (Bidinger et al., 2010; Blümmel et al., 2010; Rai et al., 2012). More importantly from this study, dual-purpose genotypes were identified and these can be used as a reference set for the developing high grain and stover yielding lines.

In the present study, a total of 11 traits were recorded for all three seasons. However, due to heavy rain, we are unable to generate quality data for the rainy season. Hence to avoid misinterpretation of results/findings, data for only six NUE traits were pooled from two summer seasons and presented. Moreover, the NPG was slightly increased with the nitrogen application and genetic differences for the traits were also observed. These results are in tune with the report of Belete et al. (2018), who found the higher NPG with increased N levels. NPG in N_0_ was reduced by 3.6 and 11.4% compared to N_50_ and N_100_, respectively. Whereas, in N_50_ condition, the mean NPG decreased by 8.24% as compared to N_100_. These results are similar to the finding of Lopez-Bellido et al. (2004), Arduini et al. (2006) who found the genetic variations among wheat genotypes for grain N concentration and increased NPG with increased N levels. In the case of the NPS, the trend was contrasting to that of NPG. Herein, NPS was slightly more in N_0_ as compared with N_50_ and N_100_ but was not up to a significant level. These results concur with the earlier reports of Belete et al. (2018) who found higher NPS with increasing N levels.

Nitrogen utilization efficiency is defined as the genotype ability to assimilate and remobilize N ultimately to produce the GY (Rengel and Graham, 1995; Fageria et al., 2008). Determination of the genetic variations in NUtE is essential for the selection of efficient genotypes and can be used further in breeding programs to develop low N tolerant material. The concept of genotypes grouping is used widely in nutrient use efficiency (Fageria and Baligar, 2003). Based on NUtE efficiency data in N_0_ versus N_100_, genotypes are classified into four groups viz., N efficient non-responsive, N efficient responsive (NER), N responsive inefficient, and N inefficient non-responsive under N_0_ and N_100_ condition, respectively. In N_0_ NUtE data, a total of 198 efficient and 176 inefficient genotypes were identified and out of 198 efficient genotypes, 58 genotypes were falling in the first desirable group which is N efficient non-responsive. These genotypes are exhibiting a progressive performance under low N. This may enable breeders to develop efficient genotypes under low input environments for pearl millet breeding activities. The remaining 140 genotypes were falling in the next most desirable group, NER and these genotypes were exhibiting a progressive response to increased N availability. The NER genotypes identified from the present investigation could be the prospective targets for selection toward the genetic improvement of pearl millet for N utilization. Interestingly, in the identified 25 NIS genotypes, 20 genotypes which are falling under N efficient responsive group were showed progressive performance in terms of efficient and responsive use of nitrogen. Genotypes with more NUtE could produce high GY per unit of N consumption (Moll et al., 1982). Various studies on NUtE have already reported that breeding for efficient genotypes under low N could be achievable with high NUtE (Fageria, 2014; He et al., 2017). The third group, responsive inefficient genotypes, can be used in breeding programs. The rest of the genotypes fall into the fourth group and these are less desirable from the NUE point of view. Interestingly, few common genotypes were identified from the non-responsive and inefficient genotypes. Overall, efficient genotypes are higher in the utilization of absorbed nutrients than inefficient genotypes. Vast genotypic variations in NUtE have been reported under field/pot screening in various wheat genotypes and other crops by several researchers (Bouchet et al., 2014; Ma et al., 2015).

The nitrogen harvest index indicates the level of efficiency of plants to use acquired total N for grain formation (He et al., 2017). In the present study, significant genotypic variation was observed for NHI at both N_0_ and N_100_ conditions. NHI was positively correlated with NUtE and GY in all three N levels. These findings concur with previous studies in oilseed rape (Ulas et al., 2013; Stahl et al., 2016) and wheat (Monostori et al., 2017).

In the current study, the Pearson correlation coefficient analysis for the GY and NUE related traits indicated that NUtE trait has the largest contribution to the GY followed by NHI and TNUp across N levels, implying that GY improvement in pearl millet could be possible by selecting genotypes that attain a higher NUtE and NHI across N levels. Similarly, a significant correlation between GY and NUtE was reported in maize, wheat, and oilseed rape (He et al., 2017; Belete et al., 2018). Fageria et al. (2010) reported that strong associations between GY and NUE related traits could be a better option for GY improvements under limited N condition. Furthermore, GY was negatively correlated with NPG and NPS across N levels. Previous studies also reported a similar significant negative correlation between grain N concentration and GY (Sinebo et al., 2004). In the present study, a strong positive correlation of DSY with NUpS and TNUp across N levels was observed indicating that more DSY was with higher NUpS and TNUp, where it was negatively correlated with NPS, NUtE, and NHI. The inverse relationship between NPS and DSY was reported in rice (Subudhi et al., 2020). Identified genetic stocks will be further utilized to carry an in-depth investigation to understand the genes and associated pathways related to NUE. Overall, important correlations identified from this study, will certainly help for the future pearl millet NUE breeding programs.

## Conclusion

Improving NUE of pearl millet is pivotal for sustainable crop growth and yield especially under low nitrogen soils. Likewise, improving crop productivity using N fertilization is important for achieving climate resilience. Nevertheless, the genetic improvement of pearl millet NUE depends on the nature and extent of variation among the germplasm. As studies on pearl millet NUE are still nascent, this study was aimed to derive morphologic and agronomical traits associated with NUE in a set of 380 diverse lines under different N levels. The first step in this research revealed extensive genetic variations across the diversity panel and mapping population parents under N_0_ and N_100_ conditions. Also, large environmental variations (including N inputs) for GY and its related NUE traits were observed. Nitrogen limitation resulted in the reduction of GY and DSY in N_0_, as compared with N_50_ and N_100_. Under low N, the best grain yielding genotypes were identified and considered as nitrogen-insensitive (NIS). Genotypes IP10820, IP17720, ICMB01222-P1, IP10379, ICMB89111-P2, IP8069, ICMB90111-P2, ICMV-IS89305, and ICMV221 (ICMV88904) proved to be the most efficient genotypes in terms of GY at low and high N levels, and indeed shows their inherent genotypic plasticity toward N application. Furthermore, the use of NIS genotypes will help in breeding N-efficient genotypes for arid and marginal agro-ecologies. Overall results suggest that genotypes with more yield and high to moderate NUtE can be used as parents for the breeding of N efficient genotypes. The lines identified from the present study, coupled with multi-omics technologies can help to identify candidate genes for NUE in pearl millet. The available genetic stocks will be useful to carry an in-depth dissection of the genes and pathways related to NUE. These contrasting genetic stocks may help to map the genome, transcriptome, proteome, and metabolome signatures of the traits leading to a better understanding of NUE in pearl millet. These current findings may also help the farmers for optimizing the use of fertilizer inputs for economic and environmentally sustainable food production.

## Data Availability Statement

The original contributions presented in the study are included in the article/Supplementary Material, further inquiries can be directed to the corresponding author/s.

## Author Contributions

RKS and RG planned and designed this research. VP, MP, SB, RR, and VT performed the field experiments, phenotyping, and analyzed the data. AR, RD, and GA carried out statistical analysis. RKS and RG interpreted the data. VP, MP, RKS, and RG wrote the manuscript. All the authors have made their contribution in editing the manuscript for publication.

## Conflict of Interest

The authors declare that the research was conducted in the absence of any commercial or financial relationships that could be construed as a potential conflict of interest.

## Footnotes

1 http://darwin.cirad.fr/darwin

## Supplementary Material

Supplementary Table 1 Details of soil properties before establishment of experimental plot.

Supplementary Table 2 Phenotypic data of 380 pearl millet genotypes for five traits (SPAD, Leaf number, Leaf area, Grain yield, and Dry stover yield) at three N levels. Mean values of over three seasons (summer 2017, rainy 2017, and summer 2018).

Supplementary Table 3 Phenotypic data of 380 pearl millet genotypes for six traits (1. N percent in grain 2. N percent in stover 3. N uptake in stover 4. Total N uptake 5. Nitrogen utilization efficiency 6. Nitrogen Harvest Index) at three N levels. Mean values of over two seasons (summer 2017 and summer 2018).

Supplementary Table 4 Agronomy (days to 50% flowering and plant height) data provided for top five NIS genotypes under N_0_.

## References

[B1] Alagarswamy G. Bidinger F. R. (1987). “Genotypic variation in biomass production and nitrogen use efficiency in pearl millet [*Pennisetum americanum* (L.) Leeke],” in *Genetic Aspects of Plant Mineral Nutrition*, eds Gabelman W. H. Loughman B. C. (Dordrecht: Springer), 281–286. 10.1007/978-94-009-3581-5_25

[B2] Anbessa Y. Juskiw P. Good A. Nyachiro J. Helm J. (2009). Genetic variability in nitrogen use efficiency of spring barley. *Crop Sci.* 49 1259–1269. 10.2135/cropsci2008.09.0566

[B3] Arduini I. Masoni A. Ercoli L. Mariotti M. (2006). Grain yield, and dry matter and nitrogen accumulation and remobilization in durum wheat as affected by variety and seeding rate. *Eur. J. Agron.* 25 309–318. 10.1016/j.eja.2006.06.009

[B4] Bandyopadhyay T. Swarbreck S. M. Jaiswal V. Gupta R. Bentley A. R. Griffiths H. (2020). Grain number and genotype drive nitrogen-dependent yield response in the C4 model *Setaria italica* (L.) P. Beauv. *bioRxiv* [Preprint]. 10.1101/2020.03.23.003004

[B5] Basava R. K. Hash C. T. Mahendrakar M. D. Kishor P. B. K. Satyavathi C. T. Kumar S. (2019). Discerning combining ability loci for divergent environments using chromosome segment substitution lines (CSSLs) in pearl millet. *PLoS One* 14:e0218916. 10.1371/journal.pone.0218916 31461465 PMC6713397

[B6] Belete F. Dechassa N. Molla A. Tana T. (2018). Effect of nitrogen fertilizer rates on grain yield and nitrogen uptake and use efficiency of bread wheat (*Triticum aestivum* L.) varieties on the Vertisols of central highlands of Ethiopia. *Agric. Food Secur.* 7 1–12. 10.1186/s40066-018-0231-z

[B7] Bidinger F. R. Blümmel M. Hash C. T. Choudhary S. (2010). Genetic enhancement for superior food-feed traits in a pearl millet (*Pennisetum glaucum* (L.) R. Br.) variety by recurrent selection. *Anim. Nutr. Feed Tech*. 10 61–68.

[B8] Blümmel M. Khan A. A. Vadez V. Hash C. T. Rai K. N. (2010). Variability in stover quality traits in commercial hybrids of pearl millet (*Pennisetum glaucum* (L.) R. Br.) and grain-stover trait relationships. *Anim. Nutr. Feed Tech*. 10 29–38.

[B9] Bouchet A. S. Nesi N. Bissuel C. Bregeon M. Lariepe A. Navier H. (2014). Genetic control of yield and yield components in winter oilseed rape (*Brassica napus* L.) grown under nitrogen limitation. *Euphytica* 199 183–205. 10.1007/s10681-014-1130-4

[B10] Chan-Navarrete R. Kawai A. Dolstra O. Van Bueren E. T. L. Van der Linden C. G. (2014). Genetic diversity for nitrogen use efficiency in spinach (*Spinacia oleracea* L.) cultivars using the Ingestad model on hydroponics. *Euphytica* 199 155–166. 10.1007/s10681-014-1186-1

[B11] Chen B. Xu K. Li J. Li F. Qiao J. Li H. (2014). Evaluation of yield and agronomic traits and their genetic variation in 488 global collections of *Brassica napus* L. *Genet. Resour. Crop Evol.* 61 979–999. 10.1007/s10722-014-0091-8

[B12] D’Andrea A. C. Casey J. (2002). Pearl millet and Kintampo subsistence. *Afr. Archaeol. Rev.* 19 147–173. 10.1023/A:1016518919072

[B13] Ertiro B. T. Beyene Y. Das B. Mugo S. Olsen M. Oikeh S. (2017). Combining ability and testcross performance of drought-tolerant maize inbred lines under stress and non-stress environments in Kenya. *Plant Breed.* 136 197–205. 10.1111/pbr.12464 28781399 PMC5518761

[B14] Fageria N. K. (2014). Nitrogen harvest index and its association with crop yields. *J. Plant Nutr*. 37 795–810. 10.1080/01904167.2014.881855

[B15] Fageria N. K. Baligar V. C. (2003). Methodology for evaluation of lowland rice genotypes for nitrogen use efficiency. *J. Plant Nutr.* 26 1315–1333. 10.1081/PLN-120020373

[B16] Fageria N. K. Baligar V. C. Li Y. C. (2008). The role of nutrient efficient plants in improving crop yields in the twenty first century. *J. Plant Nutr.* 31 1121–1157. 10.1080/01904160802116068

[B17] Fageria N. K. De Morais O. P. Dos Santos A. B. (2010). Nitrogen use efficiency in upland rice genotypes. *J. Plant Nutr.* 33 1696–1711. 10.1080/01904167.2010.496892

[B18] FAO (2019). FAO World Fertilizer Trends and Outlook to 2020. Available online at: http://www.fao.org/3/a-i6895e.pdf (accessed June 11, 2019).

[B19] Gianquinto G. Goffart J. P. Olivier M. Guarda G. Colauzzi M. Dalla Costa L. (2004). The use of hand-held chlorophyll meters as a tool to assess the nitrogen status and to guide nitrogen fertilization of potato crop. *Potato Res.* 47 35–80. 10.1007/bf02731970

[B20] Good A. G. Shrawat A. K. Muench D. G. (2004). Can less yield more? Is reducing nutrient input into the environment compatible with maintaining crop production? *Trends Plant Sci*. 9 597–605. 10.1016/j.tplants.2004.10.008 15564127

[B21] Govintharaj P. Gupta S. K. Maheswaran M. Sumathi P. Atkari D. G. (2018). Correlation and path coefficient analysis of biomass yield and quality traits in forage type hybrid parents of pearl millet. *Int. J. Pure Appl. Biosci*. 6 1056–1061. 10.18782/2320-7051.5992

[B22] Gupta S. K. Nepolean T. Sankar S. M. Rathore A. Das R. R. Rai K. N. (2015). Patterns of molecular diversity in current and previously developed hybrid parents of pearl millet [*Pennisetum glaucum* (L.) R. Br.]. *Am. J. Plant Sci*. 6 1697–1712. 10.4236/ajps.2015.611169

[B23] Hakeem K. R. Chandna R. Ahmad A. Qureshi M. I. Iqbal M. (2012). Proteomic analysis for low and high nitrogen-responsive proteins in the leaves of rice genotypes grown at three nitrogen levels. *Appl. Biochem.* 168 834–850. 10.1007/s12010-012-9823-4 22903322

[B24] Hawkesford M. J. (2017). Genetic variation in traits for nitrogen use efficiency in wheat. *J. Exp. Bot.* 68 2627–2632. 10.1093/jxb/erx079 28338945

[B25] Hawkesford M. J. Griffiths S. (2019). Exploiting genetic variation in nitrogen use efficiency for cereal crop improvement. *Curr. Opin.* 49 35–42. 10.1016/j.pbi.2019.05.003 31176099 PMC6692496

[B26] He H. Yang R. Li Y. Ma A. Cao L. Wu X. (2017). Genotypic variation in nitrogen utilization efficiency of oilseed rape (*Brassica napus*) under contrasting N supply in pot and field experiments. *Front. Plant Sci.* 8:1825. 10.3389/fpls.2017.01825 29163565 PMC5664426

[B27] Hickman J. E. Palm C. A. Mutuo P. Melillo J. M. Tang J. (2014). Nitrous oxide (N 2 O) emissions in response to increasing fertilizer addition in maize (*Zea mays L*.) agriculture in western Kenya. *Nutr. Cycling Agroecosyst.* 100 177–187. 10.1007/s10705-014-9636-7

[B28] Hirel B. Le Gouis J. Ney B. Gallais A. (2007). The challenge of improving nitrogen use efficiency in crop plants: towards a more central role for genetic variability and quantitative genetics within integrated approaches. *J. Exp. Bot.* 58 2369–2387. 10.1093/jxb/erm097 17556767

[B29] Inthapanya P. Sihavong P. Sihathep V. Chanphengsay M. Fukai S. Basnayake J. (2000). Genotype differences in nutrient uptake and utilisation for grain yield production of rainfed lowland rice under fertilized and non-fertilized conditions. *Field Crops Res.* 65 57–68. 10.1016/S0378-4290(99)00070-2

[B30] Kiran T. V. Vijayalakshmi P. Rao Y. V. Swamy K. N. Kondamudi R. Srikanth B. (2016). Effects of nitrogen limitation on antioxidant enzymes, chlorophyll content and grain yield of rice genotypes. *Asian Res. J. Agri.* 1 1–10. 10.9734/arja/2016/28503

[B31] Kitajima K. Hogan K. P. (2003). Increases of chlorophyll a/b ratios during acclimation of tropical woody seedlings to nitrogen limitation and high light. *Plant Cell Environ*. 26 857–865. 10.1046/j.1365-3040.2003.01017.x 12803613

[B32] Ladha J. K. Kirk G. J. D. Bennett J. Peng S. Reddy C. K. Reddy P. M. (1998). Opportunities for increased nitrogen-use efficiency from improved lowland rice germplasm. *Field Crops Res.* 56 41–71. 10.1016/S0378-4290(97)00123-8

[B33] Le Gouis J. Béghin D. Heumez E. Pluchard P. (2000). Genetic differences for nitrogen uptake and nitrogen utilization efficiencies in winter wheat. *Eur. J. Agron.* 12 163–173. 10.1016/S1161-0301(00)00045-9

[B34] Li F. Ai T. Zhou S. Nie J. Liu F. (2003). Influence of slow-release nitrogen fertilizers on lowland rice yield and nitrogen use efficiency. *Chin. J. Soil Sci.* 35 311–315.

[B35] Li P. Chen F. Cai H. Liu J. Pan Q. Liu Z. (2015). A genetic relationship between nitrogen use efficiency and seedling root traits in maize as revealed by QTL analysis. *J. Exp. Bot*. 66 3175–3188. 10.1093/jxb/erv127 25873660 PMC4449538

[B36] Long J. R. Wan Y. Z. Song C. F. Jian S. U. N. Qin R. J. (2013). Effects of nitrogen fertilizer level on chlorophyll fluorescence characteristics in flag leaf of super hybrid rice at late growth stage. *Rice Sci.* 20 220–228. 10.1016/s1672-6308(13)60138-9

[B37] Lopez-Bellido R. J. Shepherd C. E. Barraclough P. B. (2004). Predicting post-anthesis N requirements of bread wheat with a Minolta SPAD meter. *Eur. J. Agron.* 20 313–320. 10.1016/S1161-0301(03)00025-X

[B38] Ma B. L. Biswas D. K. Herath A. W. Whalen J. K. Ruan S. Q. Caldwell C. (2015). Growth, yield, and yield components of canola as affected by nitrogen, sulfur, and boron application. *J. Plant Nutr. Soil Sci*. 178 658–670. 10.1002/jpln.201400280

[B39] Maman N. Mason S. C. Lyon D. J. (2006). Nitrogen rate influence on pearl millet yield, nitrogen uptake, and nitrogen use efficiency in Nebraska. *Commun. Soil Sci. Plant Anal.* 37 127–141. 10.1080/00103620500406112

[B40] Manning K. Pelling R. Higham T. Schwenniger J. L. Fuller D. Q. (2011). 4500-Year old domesticated pearl millet (*Pennisetum glaucum*) from the Tilemsi Valley, Mali: new insights into an alternative cereal domestication pathway. *J. Archaeol. Sci.* 38 312–322. 10.1038/nrg2612 19584810

[B41] Mhaskar N. V. Dongale J. H. Dademal A. A. Khanvilkar S. A. (2005). Performance of scented rice varieties under different nitrogen levels in lateritic soil of Konkan. *Oryza* 42:323.

[B42] Moll R. H. Kamprath E. J. Jackson W. A. (1982). Analysis and interpretation of factors which contribute to efficiency of nitrogen utilization. *J. Agron*. 74 562–564. 10.2134/agronj1982.00021962007400030037x

[B43] Monostori I. Árendás T. Hoffman B. Galiba G. Gierczik K. Szira F. (2016). Relationship between SPAD value and grain yield can be affected by cultivar, environment and soil nitrogen content in wheat. *Euphytica* 211 103–112. 10.1007/s10681-016-1741-z

[B44] Monostori I. Szira F. Tondelli A. Arendas T. Gierczik K. Cattivelli L. (2017). Genome-wide association study and genetic diversity analysis on nitrogen use efficiency in a Central European winter wheat (*Triticum aestivum* L.) collection. *PLoS One* 12:e0189265. 10.1371/journal.pone.0189265 29283996 PMC5746223

[B45] Muchow R. C. (1998). Nitrogen utilization efficiency in maize and grain sorghum. *Field Crops Res*. 56 209–216. 10.1016/S0378-4290(97)00132-9

[B46] Namai S. Toriyama K. Fukuta Y. (2009). Genetic variations in dry matter production and physiological nitrogen use efficiency in rice (*Oryza sativa* L.) varieties. *Breed. Sci*. 59 269–276. 10.1270/jsbbs.59.269 26081539

[B47] Pramanik K. Bera A. K. (2013). Effect of seedling age and nitrogen fertilizer on growth, chlorophyll content, yield and economics of hybrid rice (*Oryza sativa* L.). *Int. J. Agron. Plant Prod.* 4 3489–3499.

[B48] Presterl T. Seitz G. Landbeck M. Thiemt E. M. Schmidt W. Geiger H. H. (2003). Improving nitrogen-use efficiency in European maize. *Crop Sci.* 43 1259–1265. 10.2135/cropsci2003.1259

[B49] Rai K. N. Blummel M. Singh A. K. Rao A. S. (2012). Variability and relationships among forage yield and quality traits in pearl millet. *Eur. J. Plant Sci. Biotechnol*. 6 118–124.

[B50] Rao I. S. Neeraja C. N. Srikanth B. Subrahmanyam D. Swamy K. N. Rajesh K. (2018). Identification of rice landraces with promising yield and the associated genomic regions under low nitrogen. *Sci. Rep.* 8:9200. 10.1038/s41598-018-27484-0 29907833 PMC6003918

[B51] Raun W. R. Johnson G. V. Westerman R. L. (1999). Fertilizer nitrogen recovery in long-term continuous winter wheat. *Soil Sci. Soc. Am. J.* 63 645–650. 10.2136/sssaj1999.03615995006300030030x

[B52] Raun W. R. Solie J. B. Johnson G. V. Stone M. L. Mullen R. W. Freeman K. (2002). Improving nitrogen use efficiency in cereal grain production with optical sensing and variable rate application. *J. Agron.* 94 815–820. 10.2134/agronj2002.8150

[B53] Rengel Z. Graham R. D. (1995). Wheat genotypes differ in Zn efficiency when grown in chelate-buffered nutrient solution. *Plant Soil* 176 307–316. 10.1007/BF00011795

[B54] Russo T. A. Tully K. Palm C. Neill C. (2017). Leaching losses from Kenyan maize cropland receiving different rates of nitrogen fertilizer. *Nutr. Cycling Agroecosyst.* 108 195–209. 10.1007/s10705-017-9852-z 33488271 PMC7745104

[B55] Sahrawat K. L. Kumar G. R. Murthy K. V. S. (2002). Sulfuric acid–selenium digestion for multi-element analysis in a single plant digest. *Commun. Soil Sci. Plan* 33 3757–3765. 10.1081/CSS-120015920

[B56] Šarčević H. Jukić K. Ikić I. Lovrić A. (2014). Estimation of quantitative genetic parameters for grain yield and quality in winter wheat under high and low nitrogen fertilization. *Euphytica* 199 57–67. 10.1007/s10681-014-1154-9

[B57] Sehgal D. Skot L. Singh R. Srivastava R. K. Das S. P. Taunk J. (2015). Exploring potential of pearl millet germplasm association panel for association mapping of drought tolerance traits. *PLoS One* 10:e0122165. 10.1371/journal.pone.0122165 25970600 PMC4430295

[B58] Sial M. A. Arain M. A. Khanzada S. H. A. M. A. D. A. D. Naqvi M. H. Dahot M. U. Nizamani N. A. (2005). Yield and quality parameters of wheat genotypes as affected by sowing dates and high temperature stress. *Pak. J. Bot*. 37 575–584.

[B59] Sinebo W. Gretzmacher R. Edelbauer A. (2004). Genotypic variation for nitrogen use efficiency in Ethiopian barley. *Field Crops Res.* 85 43–60. 10.1016/S0378-4290(03)00135-7

[B60] Singh T. Shivay Y. S. Singh S. (2004). Effect of date of transplanting and nitrogen on productivity and nitrogen use indices in hybrid and non-hybrid aromatic rice. *Acta Agron. Hung*. 52 245–252. 10.1556/aagr.52.2004.3.5

[B61] Srikanth B. Rao I. S. Surekha K. Subrahmanyam D. Voleti S. R. Neeraja C. N. (2016). Enhanced expression of OsSPL14 gene and its association with yield components in rice (*Oryza sativa*) under low nitrogen conditions. *Gene* 576 441–450. 10.1016/j.gene.2015.10.062 26519999

[B62] Srivastava R. Singh R. B. Lakshmi P. V. Srikanth B. Madhu P. Satyavathi C. T. (2019). Genome-wide association studies (GWAS) and genomic selection (GS) in pearl millet: advances and prospects. *Front. Genet*. 10:1389. 10.3389/fgene.2019.01389 32180790 PMC7059752

[B63] Stahl A. Friedt W. Wittkop B. Snowdon R. J. (2016). Complementary diversity for nitrogen uptake and utilisation efficiency reveals broad potential for increased sustainability of oilseed rape production. *Plant Soil* 400 245–262. 10.1007/s11104-015-2726-8

[B64] Subudhi H. N. Prasad K. V. S. V. Ramakrishna C. Rameswar P. S. Pathak H. Ravi D. (2020). Genetic variation for grain yield, straw yield and straw quality traits in 132 diverse rice varieties released for different ecologies such as upland, lowland, irrigated and salinity prone areas in India. *Field Crops Res.* 245:107626 10.1016/j.fcr.2019.107626

[B65] Tako E. Reed S. M. Budiman J. Hart J. J. Glahn R. P. (2015). Higher iron pearl millet (*Pennisetum glaucum* L.) 967 provides more absorbable iron that is limited by increased polyphenolic content. *Nutr. J*. 14 1–9. 10.1186/1475-2891-14-11 25614193 PMC4325945

[B66] Ulas A. Behrens T. Wiesler F. Horst W. J. (2013). Does genotypic variation in nitrogen remobilisation efficiency contribute to nitrogen efficiency of winter oilseed-rape cultivars (*Brassica napus* L.). *Plant Soil* 371 463–471. 10.1007/s11104-013-1688-y

[B67] Vijayalakshmi P. Kiran T. V. Rao Y. V. Srikanth B. Rao I. S. Sailaja B. (2013). Physiological approaches for increasing nitrogen use efficiency in rice. *Indian J. Plant Physiol*. 18 208–222. 10.1007/s40502-013-0042-y

[B68] Vijayalakshmi P. Vishnukiran T. Kumari B. R. Srikanth B. Rao I. S. Swamy K. N. (2015). Biochemical and physiological characterization for nitrogen use efficiency in aromatic rice genotypes. *Field Crops Res.* 179 132–143. 10.1016/j.fcr.2015.04.012

[B69] VSN International (2019). *Genstat for Windows*, 20th Edn Hemel Hempstead: VSN International.

[B70] Worku M. Bänziger M. Erley G. S. A. M. Friesen D. Diallo A. O. Horst W. J. (2007). Nitrogen uptake and utilization in contrasting nitrogen efficient tropical maize hybrids. *Crop Sci*. 47 519–528. 10.2135/cropsci2005.05.0070

[B71] Würschum T. (2012). Mapping QTL for agronomic traits in breeding populations. *Theor. Appl. Genet.* 125 201–210. 10.1007/s00122-012-1887-6 22614179

[B72] Yang H. Yang J. Lv Y. He J. (2014). SPAD values and nitrogen nutrition index for the evaluation of rice nitrogen status. *Plant Pro. Sci.* 17, 81–92. 10.1626/pps.17.81

